# A PRDM16-driven signal regulates body composition in testosterone-treated hypogonadal men

**DOI:** 10.3389/fendo.2024.1426175

**Published:** 2024-09-02

**Authors:** Siresha Bathina, Georgia Colleluori, Dennis T. Villareal, Lina Aguirre, Rui Chen, Reina Armamento-Villareal

**Affiliations:** ^1^ Division of Endocrinology Diabetes and Metabolism at Baylor College of Medicine, Houston, TX, United States; ^2^ Department of Medicine, Michael E. DeBakey Veterans Affairs (VA) Medical Center, Houston, TX, United States; ^3^ Department of Medicine, University of New Mexico School of Medicine, Albuquerque, NM, United States; ^4^ Department of Medicine, New Mexico VA Health Care System, Albuquerque, NM, United States

**Keywords:** adipogenesis, myogenesis, PRDM16, estrogen, testosterone

## Abstract

**Background:**

Testosterone (T) therapy increases lean mass and reduces total body and truncal fat mass in hypogonadal men. However, the underlying molecular mechanisms for the reciprocal changes in fat and lean mass in humans are not entirely clear.

**Methods:**

Secondary analysis of specimens obtained from a single-arm, open-label clinical trial on pharmacogenetics of response to T therapy in men with late-onset hypogonadism, conducted between 2011 and 2016 involving 105 men (40-74 years old), who were given intramuscular T cypionate 200 mg every 2 weeks for 18 months. Subcutaneous fat (SCF), peripheral blood mononuclear cells (PBMC) and serum were obtained from the participants at different time points of the study. We measured transcription factors for adipogenesis and myogenesis in the SCF, and PBMC, respectively, by real-time quantitative PCR at baseline and 6 months. Serum levels of FOLLISTATIN, PAX7, MYOSTATIN, ADIPSIN, and PRDM16 were measured by ELISA.

**Results:**

As expected, there was a significant increase in T and estradiol levels after 6 months of T therapy. There was also a reduction in fat mass and an increase in lean mass after 6 months of T therapy. Gene-protein studies showed a significant reduction in the expression of the adipogenic markers *PPARγ* in SCF and ADIPSIN levels in the serum, together with a concomitant significant increase in the expression of myogenic markers, *MYOD* in PBMC and PAX7 and FOLLISTATIN levels in the serum after 6 months of T therapy compared to baseline. Interestingly, there was a significant increase in the adipo-myogenic switch, *PRDM16*, expression in SCF and PBMC, and in circulating protein levels in the serum after 6 months of T therapy, which is likely from increased estradiol.

**Conclusion:**

Our study supports that molecular shift from the adipogenic to the myogenic pathway in men with hypogonadism treated with T could be mediated directly or indirectly by enhanced PRDM16 activity, in turn a result from increased estradiol level. This might have led to the reduction in body fat and increase in lean mass commonly seen in hypogonadal men treated with T.

## 1 Introduction

Age-related reduction in gonadal steroids is associated with changes in body composition, i.e., increase in fat mass and decrease in lean mass (1) These body composition changes, result not only from the lack of androgens, but also from the reduced amount of the androgen-derived estrogen (2). In these subjects, testosterone (T) therapy results in improvement in the body composition (3) possibly due to the combined action of T and that of estradiol (E2) deriving from T. Previous studies from our lab demonstrated the improvement in body composition with T therapy (4, 5). The mechanism underlying the effect of T in enhancing myogenesis and ablating adipogenesis has been explored *in vitro* (6), and in pre-clinical models (7) however, data in humans are scarce. Myocytes, and adipocytes originate from a common mesenchymal stem cell (MSCs) (8). How MSCs differentiate into distinct lineages under the influence of T in hypogonadal men needs to be explored. The commitment of MSCs to a particular lineage always rely on interaction among transcriptional regulators with crucial genes. One such gene is positive regulatory domain zinc finger region protein 16 (PRDM16),a highly conserved 140kDa zinc finger transcriptional co-regulator (9) which has important function in cell fate determination and function of brown and beige adipocytes, in maintenance of hematopoietic and neural stem cells, and proliferation of cardiomyocytes (10, 11). Though PRDM16 is a nuclear bound transcription factor, Pinheiro et al, identified Prdm16 and Prdm3 as redundant H3K9ME1-speciifc methyl transferases in the cytoplasm directing methylation via anchoring to nuclear periphery to maintain integrity of mammalian heterochromatin (12). Evidence suggests that Prdm16, transcriptional factor involved in brown fat adipogenesis (13), interacts with peroxisome proliferator-activated receptor γ (PPARγ) (14) and CCAAT-enhancer binding protein-alpha (CEBPα) (15) resulting in the trans-differentiation of myoblasts to brown adipocytes (16). When it comes to interaction between these genes and sex steroids, Zhao et al. found that androgen receptor (AR) directly binds the *Prdm16* locus and inhibit white adipose tissue (WAT) browning via suppression of *Prdm16.* These investigators also reported higher PRDM16 levels in WAT of women (17). Moreover, the administration of E2 resulted in WAT browning from an increase in the browning genes *UCP-*1, *PGC-1α*, and *Prdm16* and decreased body weight and visceral fat in ovariectomized (OVX) mice (18). Altogether, the above studies imply the crucial role of PRDM16 in adipose tissue biology. Furthermore, though the above findings are consistent with the regulatory role of both T and E2 in adipose tissue deposition and browning, the specific factors involved are still to be identified.

Aside from prior study showing that T administration in eugonadal men resulted in an increase in PAX7 (Paired box 7), a transcription factor which regulates the regeneration and proliferation of myogenic precursors (19) most of the information available comes from *in-vitro* or pre-clinical models with limited scope. In this study, we aim to evaluate the *in-vivo* gene-protein machinery involved in adipogenesis and myogenesis in hypogonadal men given T therapy. We hypothesize that PRDM16 plays a crucial role in the observed reduction in fat mass and increase in lean mass with T therapy in hypogonadal men. Results from this study may provide a consolidated mechanistic insight on how T improves the body composition in hypogonadal men.

## 2 Materials and methods

### 2.1 Study design, study participants, and intervention

This study is an analysis of the longitudinal data and samples obtained from a prior open-label clinical trial (NCT01378299) investigating the pharmacogenetics of CYP19A1 gene on the response to testosterone therapy in men with hypogonadism. A total of 342 male veterans attending the Endocrine, Urology and Primary Care Clinics of the New Mexico Veterans Administration Health Care System (NMVAHCS) and Michael E. DeBakey Veterans Affairs Medical Center (MEDVAMC) were screened for the study. Recruitment was accomplished either through flyers or letters to physicians about patients who may qualify for the study. Written informed consent was obtained from each subject. The protocol was approved by the Institutional Review Board of the University of New Mexico School of Medicine and the Baylor College of Medicine; and study was conducted in accordance with guidelines in the Declaration of Helsinki for the ethical treatment of human subjects.

### 2.2 Inclusion, and exclusion criteria

Information regarding study design, inclusion, and exclusion criteria of the subjects, as well as details of T therapy have been published elsewhere (4), Briefly, participants in this study included men, between 40 and 75 years of age with an average fasting, morning (between 8 to 11AM) total T level taken twice, at least 30 minutes apart, of <300 ng/dl, with no medical problems that may prevent them from finishing the study. Exclusion criteria included treatment with bone-acting drugs (e.g., bisphosphonates, teriparatide, denosumab, glucocorticoids, sex steroid compounds, selective estrogen receptor modulators, androgen deprivation therapy, and anticonvulsants) and finasteride. Additional exclusion criteria included osteoporosis and history of fragility fractures or diseases known to affect bone metabolism, such as hyperparathyroidism, chronic liver disease, uncontrolled or untreated hyperthyroidism, and significant renal impairment (creatinine of >1.5 mg/dl). Those with a history of prostate cancer, breast cancer, and untreated sleep apnea also met the criteria for exclusion.

### 2.3 Testosterone therapy

Testosterone cypionate was initiated at a dose of 200 mg every 2 weeks by intramuscular injection and adjusted to a target serum testosterone level between 17.3 to 27.2 nmol/L (500–800 ng/dl). However, after the 3rd year of the study upon the direction of the FDA, this target was changed to 17.3–20.8 nmol/L (300–600 ng/dl). This change affected the data in the last 6M of 16 subjects at NMVAHCS and all 15 subjects at MEDVAMC. Comparing testosterone levels at different timepoints showed no significant differences between those who were and those not affected by the change except at 6M where levels are higher for those affected. T therapy was given for 18 months. Fifty-one participants at NMVAHCS did self-injections, 38 received injections from the study team only, while 2 started with the study team and later did self-injections. At MEDVAMC, 5 subjects received injections from the study team while 10 did self-injections. Dosage adjustments were based on serum testosterone and hematocrit levels, and occurrence of symptoms and done by increments or decrements of 50 mg. A decrease in the dose was done for patients who develop a hematocrit of >52%. Repeat testosterone measurement was performed 2 months after a dose change, including a repeat hematocrit for those with elevated hematocrit. Otherwise, testosterone levels were measured at baseline, 3, 6, 12 and 18 months. Safety monitoring included assessment of prostate specific antigen (PSA), hematocrit, lipid profile, liver enzymes at baseline, 3, 6, 12, and 18 months.

### 2.4 Body mass index (BMI)

Body mass index (BMI) calculated as weight (kg) divided by the square of the height (m2). Height and weight were measured using a standard stadiometer and weighing scale, respectively.

### 2.5 Body composition

Assessment of body composition was performed by dual-energy X-ray absorptiometry (DXA) (Hologic-Discovery; Enhanced Whole Body 11.2 software version; Hologic Inc, Bedford, MA; USA) at baseline, 6, 12 and 18 months as previously described (4) Fat-free mass was calculated by adding whole body bone mineral content to the lean mass. The CV for lean mass and fat mass in our laboratory is 1.5% (20).

### 2.6 Subcutaneous fat biopsy

Fat biopsies were performed on patients recruited at the NMVAHCS at baseline and after 6 months of T therapy as previously described (4). Briefly, fat samples were obtained from the abdominal subcutaneous adipose tissue depot. The periumbilical area was disinfected and local anesthesia using 2% lidocaine was applied before proceeding with a small incision. Adipose tissue (~500 mg) was collected using a liposuction needle under sterile conditions. Samples were then washed in isotonic NaCl, snap-frozen in liquid nitrogen and kept at −80°C until utilization for the gene expression studies. A limited number of samples were available to perform longitudinal analysis of gene and protein expression that occur with T therapy in hypogonadal men (N=15 for SAT, N=22 for PBMC and N=38 for serum assessments).

### 2.7 Gene expression studies

#### 2.7.1 Subcutaneous fat (SCF)

We measured the adipogenic transcription factors (*PPARγ*, *CEBPα*), enzymes (*LPL*, Adipsin) from (SCF) in triplicates using real-time quantitative polymerase chain reaction at BL and 6M respectively. Total RNA was isolated from SCF obtained at baseline and after 6M using RNeasy Plus Universal Mini Kit (QIAGEN, Valencia, CA, USA) and Fast Prep 24–5G homogenizer (MP Biomedicals, Santa Ana, CA, USA) following the manufacturer’s instructions. The quality and quantity of total RNA were analyzed by using both, nanodrop and Bioanalyzer 2100 (Agilent Technologies, Santa Clara, CA, USA). RNA was extracted as above and 200ng of RNA were used for retro transcription into cDNA and performed using Superscript VILO Master Mix (Invitrogen, Carlsbad, CA, USA) following protocol instructions. FAM-labeled TaqMan Gene expression assays (Applied Biosystem, College Station, TX, USA) for *PPARγ* (Assay ID: Hs01115513_m1); *CEBPα* (Assay ID: Hs00269972_s1); Lipoprotein Lipase *(LPL)*(Assay ID: Hs00173425_m1), and VIC-labeled TaqMan gene expression assay for housekeeping 18S (Assay ID: Hs03928990_g1) and TaqMan Universal Master Mix in triplicates were used following the manufacturer’s protocol. Estrogen receptor (ESRα) and Cytochrome P450 Family 19 Subfamily A member 1 (*CYP19A1*) gene expression from SCF were previously analyzed and reported in a prior publication (20) The gene expression data of both genes in the subset of men who were part of this study are included in the analysis.

#### 2.7.2 Peripheral blood mononuclear cells (PBMC)

*PRDM16, MYF-5, MYO-D* and *PAX-7* gene expression from PBMC was performed by real-time quantitative polymerase chain reaction at baseline (BL), 6months (6M). As earlier results from our lab indicated that the effect of T therapy is maximal after 6M, we chose to study the specimens at BL and 6M. RNA was extracted from PBMC using RiboPure Blood (Invitrogen, #AM1928). 200ng of RNA were used for retro transcription into cDNA and performed using Superscript VILO Master Mix (Invitrogen, Carlsbad, CA, USA) in triplicates following protocol instructions. FAM-labeled TaqMan Gene expression assays (Applied Biosystem, College Station, TX, USA) for myoblast determination protein 1 (*MYO*D1) (Assay ID: Hs00159528_m1); Myogenic factor 5 (*MYF5)* (Assay ID: Hs00929416_g1); *PRDM16* (Assay ID: Hs00223161_m1); *PAX7* (Assay ID: Hs00242962_m1); and VIC-labeled TaqMan gene expression assay for housekeeping 18S (Assay ID: Hs03928990_g1) and TaqMan Universal Master Mix were used following the manufacturer’s protocol.

#### 2.7.3 Relative quantification

ΔΔCT relative quantification: gene expression of our sample vs gene expression of human control total RNA (Applied Biosystem #4307281) adjusted for housekeeping gene expression. Data analysis was performed using Real Time PCR system QuantStudio5 and Quant Studio Design & Analysis Software 1.3.1, respectively.

### 2.8 Elisa studies

The following were measured using enzyme linked immunosorbent assay (ELISA)kits: Human ADIPONECTIN kit EZHADP-61K Human Leptin kit, EZHL-805K, Temecula, CA, USA); Human FOLLISTATIN (FST) ELISA kit, DFN00; Human GDF-8/MYOSTATIN Immunoassay DGDF80, (Quantikine; R&D Systems, Minneapolis, MN,USA),; Human CFD Elisa kit (EHCFD) (Invitrogen, Carlsbad, CA, USA), PRDM16 Human Elisa kit (Biomatik, EKN52948, Wilmington, Delaware, USA); PAX7 Immunoassay kit (MBS2606278). The CVs for the above assays in our laboratory are <10%. There were a limited number of patients who had corresponding baseline and 6-month samples available to perform longitudinal analysis of gene and protein expression, i.e., N=15 for SCF, N=22 for PBMC and N=38 for serum assessments. An additional file shows the details of kits, chemicals, and instrumentation details (See **Supplementary Table**) used in this study.

### 2.9 Statistical analysis

As earlier results from our lab, effect of T therapy is maximal after 6M, we chose to study the specimens at baseline (BL) and 6months (6M). All data are presented as mean ± SEM in figures and mean ± SD in table, were analyzed by Two-tailed Student’s paired or unpaired t test. The association between changes in body composition and changes in PRDM16 were analyzed by simple regression analysis. All analyses were performed using Prism 9.0 (GraphPad, San Diego, CA, USA). A p value of < 0.05 is considered significant.

## 3 Results

**Table 1** shows the body composition, hormonal profile, adipogenic and myogenic markers in the forty men with available baseline (BL) and 6M samples of SCF, PBMC and serum included in the analysis. There were significant increase in T (BL: 258.6 ± 90.34 ng/dl vs 6M:578.3 ± 241.7 ng/dl, p=0.001), E2 (BL: 15.69 ± 6.06 pg/ml vs 6M:39.4 ± 21.2 pg/ml, p=0.001) and estradiol/testosterone ratio (E/T) (BL: 0.70 ± 90.38 vs 6M:71.3 ± 40, p=0.001) (**Table 1**) at 6 months when compared to baseline. Furthermore, T therapy resulted in a significant decrease in total % body fat (BL:30.71 ± 6.35% vs 6M:27.8 ± 5.2%, p<0.05) along with significant increase in total lean mass (BL: 63201 ± 5894 g vs 6M: 67006 ± 7495 g, p=0.03) (**Table 1**) There were no significant differences in the other parameters such appendicular lean mass, trunk fat mass, total body fat mass and fat free mass between BL and 6 months.

**Table 1:** Six months of T therapy improved the body composition in hypogonadal men.

Parameter in Study-	Baseline (BL) characteristics	T Therapy (6M)	P value
Age	59.8 ± 8.5	60.1 ± 8.7	0.86
BMI	32.1 ± 5.1	32.2 ± 4.9	0.90
Testosterone	258.6 ± 90.34	578.3 ± 241.7	**0.001**
Estradiol	15.69 ± 6.06	37.39 ± 21.2	**0.001**
E/T	0.70 ± 0.38	71.3 ± 40.1	**0.001**
Total body fat mass (g)	31505 ± 11391	28222 ± 9629	0.21
Total % body fat	30.71 ± 6.35	27.79 ± 5.2	**<0.05**
Trunk fat mass (g)	16843 ± 7495	14921 ± 6227	0.27
Total lean mass (g)	63201 ± 5894	67006 ± 7495	**0.03**
Appendicular lean mass (g)	29043 ± 4474	30586 ± 4040	0.14
Fat-free mass (g)	67696 ± 7652	69669 ± 7637	0.29
Leptin (ng/ml)	2.7 ± 2.01	2.1 ± 1.5	0.15
Adiponectin (µg/ml)	49.7 ± 27.6	45.37 ± 27.1	0.50
*CYP19A1-Fat*	2.99 ± 0.90	6.58 ± 2.26	0.26
*ESRα-Fat*	56.2 ± 17.76	68.96 ± 28.8	0.74
ADIPSIN (ng/ml)	1864 ± 1538	1193 ± 520.5	**0.02**
PRDM16 (ng/ml)	0.30 ± 0.11	0.55 ± 0.72	**<0.05**
PAX7 (ng/ml)	31.2 ± 13.3	42.3 ± 13.9	**0.001**
FOLLISTATIN (pg/ml)	2344 ± 1684	3508 ± 1725	**0.003**
MYOSTATIN (pg/ml)	3437 ± 2029	2882 ± 1800	0.40

The above table explore the Baseline and 6M characteristics of the subset who participated in the study. Six months of Testosterone (T) therapy significantly enhanced T, estradiol, E/T along with serum levels of Myogenic (PRDM16, PAX7, FST) and total lean mass, while decreased total % body fat, adipokine ADIPSIN. BMI, Body mass index; E, estradiol; T, Testosterone; CYP19A1 gene, Cytochrome P450 Family 19 Subfamily A member 1; ESRα gene, Estrogen receptor alpha; PAX7, Paired box7 protein; PRDM16, PR domain containing protein 16; The bolded p values are statistically significant, All values are Mean ± SD.

We next studied the gene and protein machinery that may be involved in the changes in body composition we observed *in-vivo*.

### 3.1 Adipogenic

**Figure 1:**
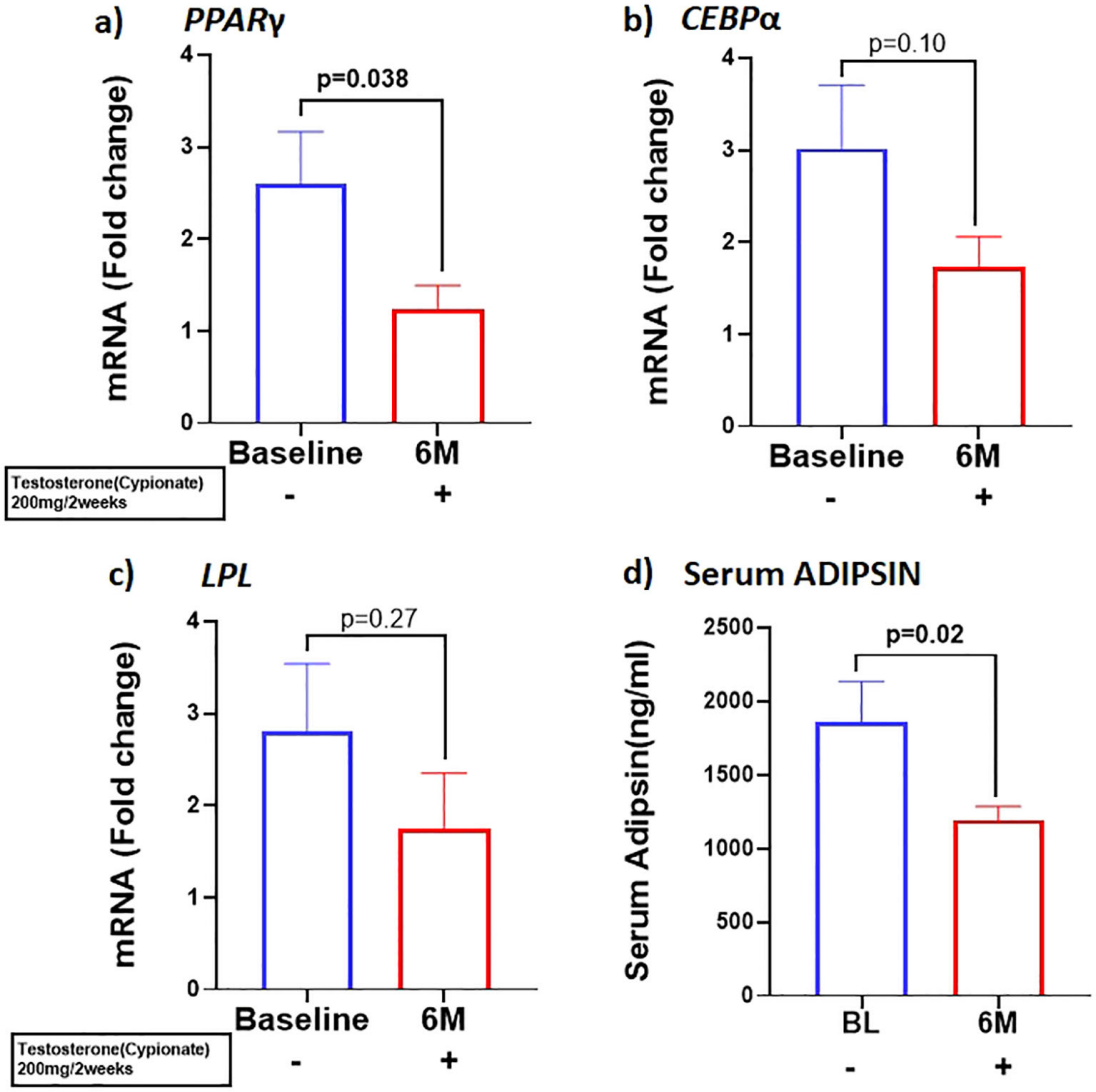
T therapy downregulated the adipogenic gene machinery with reduction in mRNA levels of adipogenic markers **(A)** *PPARγ*, **(B)** *CEBPα*, and **(C)** *LPL* in Sub cutaneous fat (SCF), and in **(D)** Adipsin protein levels in the serum after 6 months of T-therapy **i**n hypogonadal men. Data shown as Mean ± SEM. All analyses were done using Two-tailed Student’s paired or unpaired t test; bolded p values are significant at 6 months compared to baseline.

Our results showed that T therapy resulted in the significant reduction in the adipogenic gene mRNA levels of *PPARγ*, in SCF (BL: 2.59 ± 0.57 vs 6M: 1.22 ± 0.26, p=0.03) (**Figure 1A**) Gene expression of *CEBPα*, displayed a non-significant trend for reduction (1.7-fold from baseline; p=0.10) (**Figure 1B**) in SCF. Similarly, *LPL*, a key factor in lipid homeostasis, was also non-significantly reduced (BL: 2.81 ± 0.73 vs 6M: 1.74 ± 0.6, p=0.27) (**Figure 1C**). On the other hand, the levels of serum ADIPSIN, significantly decreased with T therapy (BL:1864 ± 1538 ng/ml vs 6M:1193 ± 520.5 ng/ml p=0.02) (**Figure 1D**), while levels of serum ADIPONECTIN and LEPTIN did not vary significantly (**Table 1**).

### 3.2 Myogenic

**Figure 2:**
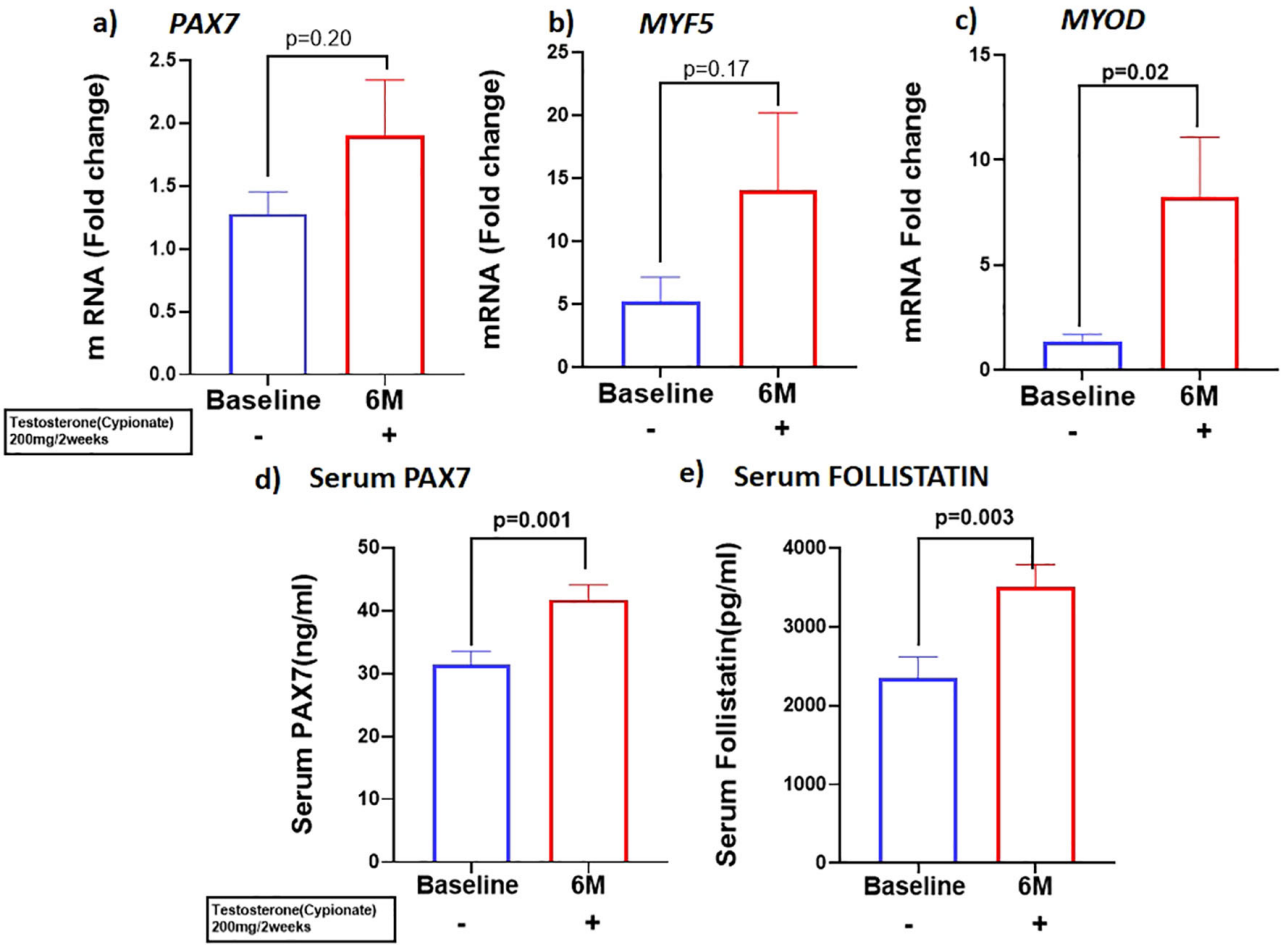
T therapy enhanced Myogenic gene machinery. mRNA levels of **(A)** *PAX7* and **(B)** *MYF5* were moderately increased along with significant increase in mRNA levels of myogenic marker **(C)** *MYOD* in peripheral blood mononuclear cells (PBMC); Protein levels of **(D)** Serum PAX7 and **(E)** Serum FOLLISTATIN after 6 months of T-therapy in hypogonadal men. Data shown as Mean ± SEM. All analyses were done using Two-tailed Student’s paired or unpaired t test; bolded p values are significant at 6 months compared to baseline.

Because previous studies have demonstrated that PBMC gene expression is strongly correlated with skeletal muscle transcriptional profile (21) we examined the expression of skeletal muscle gene machinery in PBMC. There was a non-significant trend for increased expression of *PAX7* with T therapy (BL:1.27 ± 0.17 vs 6M: 1.89 ± 0.44, p=0.2) (**Figure 2A**), in PBMC. However, a significant increase in PAX7 from baseline (BL:31.22 ± 13.3 ng/ml vs 6M: 42.3 ± 13.9 ng/ml, (p=0.001) (**Figure 2D**) was observed in the serum. Because Pax7 regulates the activation of myogenic regulators (22), we next analyzed the expression of the myogenic lineage gene machinery by RT-qPCR in PBMC. Although T therapy did not result in a significant increase in expression of the myomarker *MYF5* (BL: 6.1 ± 9.7 vs 6M: 14.06 ± 6.14, p=0.18), (**Figure 2B**), there was a significant increase in the expression of its downstream effector *MYOD1*, (**Figure 2C**) (BL:1.34 ± 0.35 vs 6M: 8.2 ± 12.7, p=0.02). Also, previous studies found that T-therapy upregulated FST in satellite cells (23), hence we assessed FST serum levels following 6M of T therapy and observed a significant increase (BL: 2344 ± 1684 pg/ml vs 6M: 3508 ± 1745 pg/ml, p=0.003) (**Figure 2E**) in levels. However, serum MYOSTATIN levels did not vary following T treatment (BL: 3437 ± 2029 pg/ml vs 6M: 2882 ± 1800pg/ml, p=0.40) (**Table 1**).

### 3.3 Adipomyogenic switch

**Figure 3:**
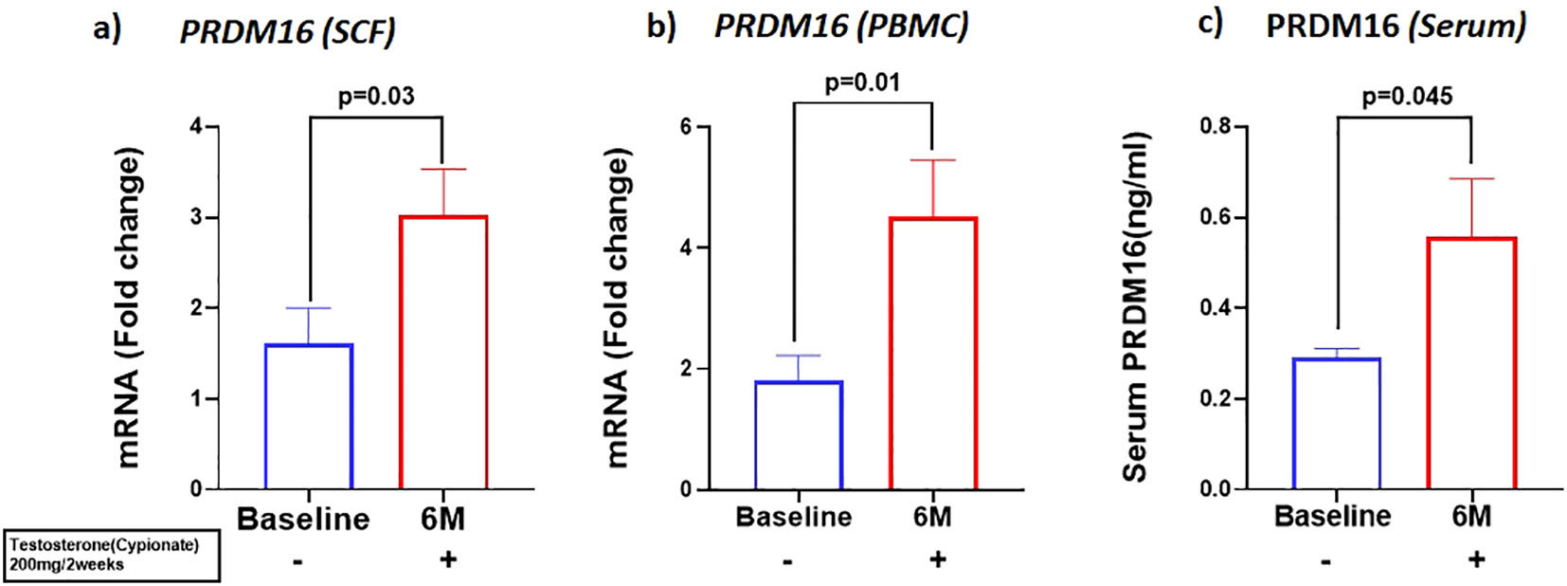
Increase in the expression of PRDM16, in mRNA levels of **(A)** *PRDM16* in Sub cutaneous fat (SCF) and **(B)** *PRDM16* in peripheral blood mononuclear cells (PBMCs), and **(C)** Protein levels (Serum PRDM16) after 6 months of T-therapy in hypogonadal men. Data shown as Mean ± SEM. All analyses were done using Two-tailed Student’s paired or unpaired t test; bolded p values are significant at 6 months compared to baseline.

We investigated the expression of the adipo-myogenic switch, PRDM16, and observed a significant upregulation of *PRDM16* expression in the SCF (BL: 2.59 ± 0.56 vs 6M: 1.23 ± 0.26, p=0.03). (**Figure 3A**) and in the PBMC (BL: 1.8 ± 0.42 vs 6M: 4.5 ± 0.92, p<0.01), (**Figure 3B**) after 6 months of T therapy. Serum level of PRDM16 was also significantly increased (BL: 0.30 ± 0.11 ng/ml vs 6M: 0.55 ± 0.72 ng/ml, p=0.042) (**Figure 3C**) following 6M of T therapy.

**Figure 4:**
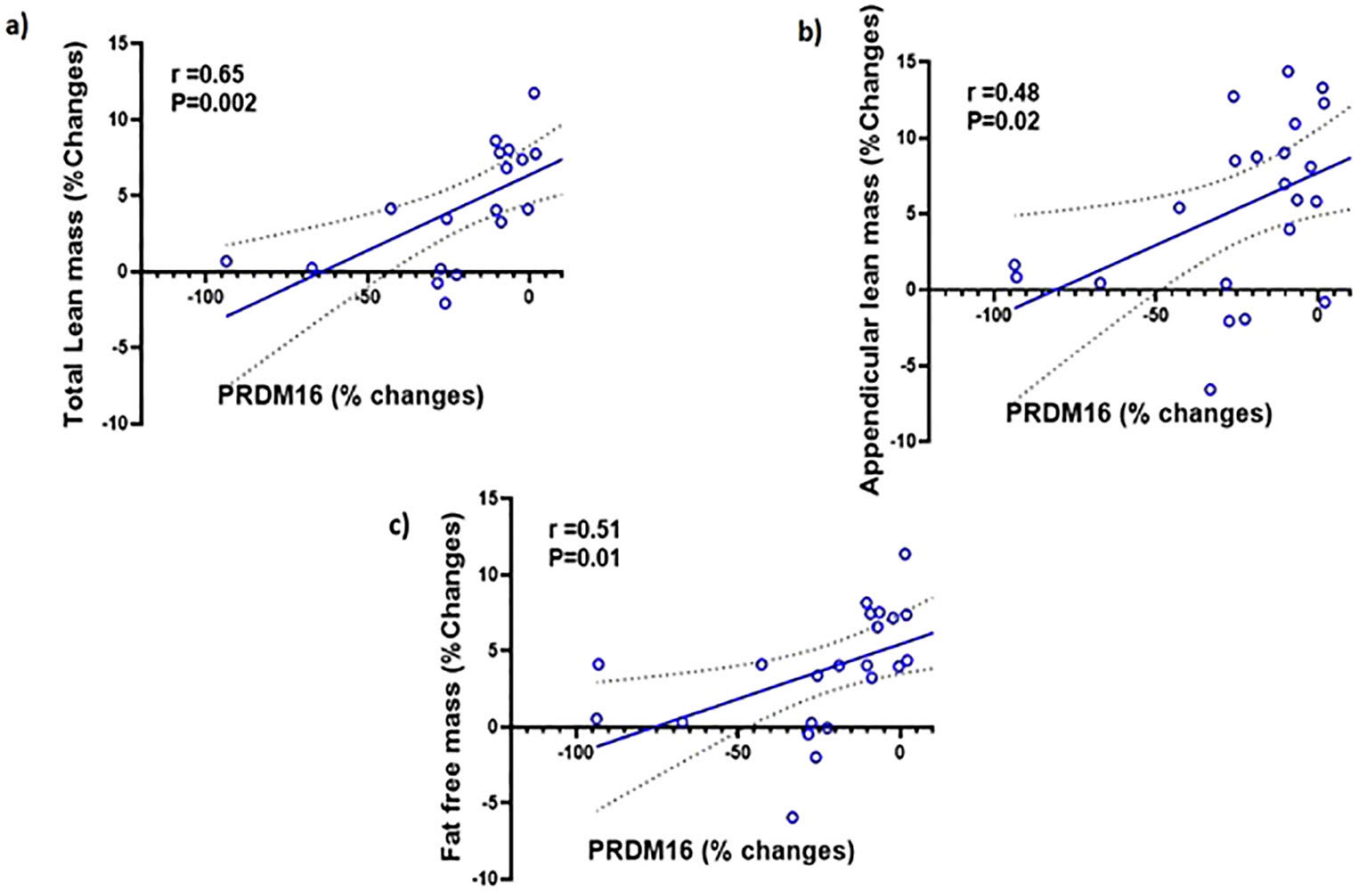
Simple regression analysis showing changes (%) in PRDM16 in the serum are significantly positively correlated with changes (%) in: **(A)** total lean mass (r=0.65, P=0.002); **(B)** Appendicular lean mass (r=0.48, P=0.02); **(C)** fat-free mass (r=0.51, P=0.01) with testosterone therapy.

We examined the correlation between the changes PRDM16 levels in the different tissues and body composition. We found no significant correlations between the changes in PRDM16 in the SCF, PBMC and serum with changes in any parameters of body fat. We also found no correlation between the changes in PRDM16 in the SCF and PBMC with total and appendicular lean and fat-free mass. However, significant correlations were observed between changes in PRDM16 protein levels in the serum with changes in total lean mass (r=0.65, p=0.002), appendicular lean mass (r=0.48, p=0.02) and fat-free mass (r=0.51, p=0.01), see **Figure 4**.

## 4 Discussion

Previous studies demonstrated a reduction in total body and truncal fat mass along with an increase in lean mass in T-treated hypogonadal men (4, 5). Although animal and *in-vitro* studies suggest reduced adipogenesis coupled with increased myogenesis as the mechanisms for this observation, occurrence of these changes in humans and which pathways are involved, remain uncertain. The present study confirmed the reduction in the expression of adipogenic and an increase in myogenic modulators occur in human subjects taking T. More importantly, our results also suggest for the first time the potential role of enhanced PRDM16 in regulating these pathways perhaps directly or indirectly by unknown downstream targets which was never explored before, providing insights into a regulatory network that underpins the positive effects of T on body composition in men with hypogonadism.

**Figure 5:**
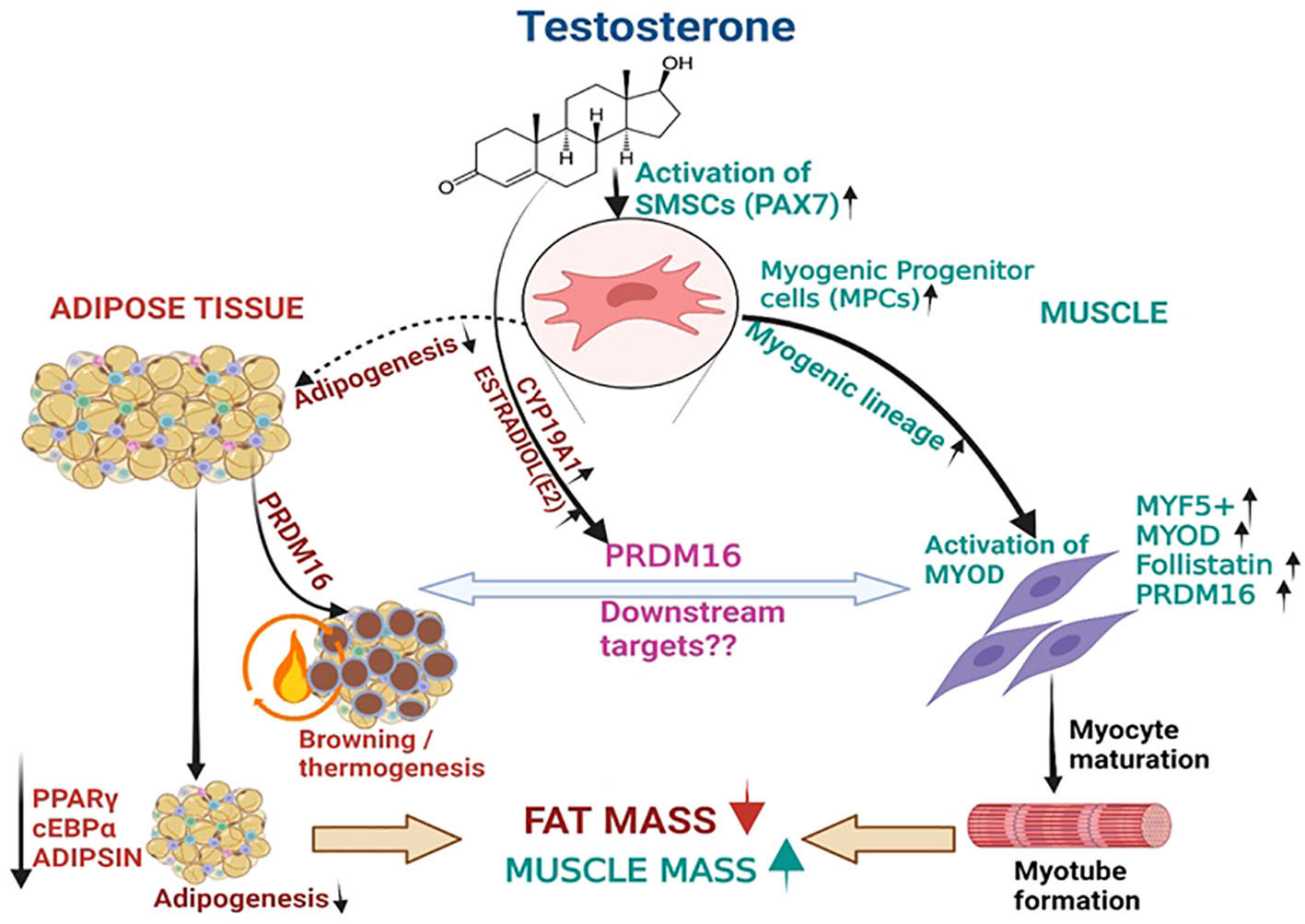
T therapy reduced adipogenic machinery and enhanced Myogenic gene machinery. T activate the MSCs via PAX7 to promote the commitment of MSCs to the myogenic lineage and subsequently activate MYF5 a key transcription factor for myogenic stem cells commitment to myoblast lineage. This in turn might increase the expression of its downstream effector myoblast determination protein 1 (*MYOD1)*, a transcription factor promoting myoblast proliferation and FOLLISTATIN leading to myotube formation. On the other hand, Estradiol formed from T by the action of CYP19A1 activates the formation of PRDM16, which not only acts as a bi-directional switch between adipogenesis and myogenesis but also causes thermic browning which along with unknown downstream targets might enhance myogenesis. Thus, adipogenic gene machinery of PPARγ, and ADIPISIN are decreased significantly leading to reduction in fat mass by T therapy.

The anti-adipogenic effect of androgens has been suggested by studies demonstrating that T and its metabolite, dihydroxytestosterone (DHT), downregulate *PPARγ*, a key transcription factor regulating adipogenesis and *CEBPα*, a transcription factor that coordinates proliferation and differentiation of adipocytes (24). This results in inhibition of the commitment of subcutaneous human adipose stem cells obtained from nonobese women to preadipocytes and reduce early-stage adipocyte differentiation Moreover, Singh et al., reported that administration of T (0-300nM) or DHT(0-30nM) to C3H10T1/2 to pluripotent cells not only inhibited adipogenic lineage, but also promoted the commitment of MSCs to the myogenic lineage (6). On the other hand, the CYP19A1 activity present in the adipose tissue results in conversion of T to E2 (See **Figure 5**). *In vivo* studies in female ovariectomized rats demonstrated downregulation of the adipogenic markers with E2 treatment (25). These studies suggest that both T and E2 are involved in downregulation of adipogenesis. Our results agree with these findings; we observed a significant reduction in adipogenic machinery, primarily *PPARγ*, in SCF following 6M of T therapy. Moreover, even though we did not detect significant changes in circulating LEPTIN and ADIPONECTIN, we found significant decrease in ADIPSIN, another adipokine, linked to increased fat mass and adipose tissue dysfunction in metabolic disorders (26), at 6M of T therapy. Since myocytes and adipocytes are derived from the same MSCs, we sought to elucidate the T-induced signaling that may explain the fat and muscle changes observed in hypogonadal men.

PRDM16 is recognized as the factor involved in the switch from white to brown adipocytes, and possibly also as the interconvertible switch between myocyte to adipocyte lineage differentiation (16). Interestingly, we found a significant increase in PRDM16 expression in SCF, PBMC, and in serum with T therapy. We believe that this is due to the increase in E2 which likely upregulates PRDM16 in the different tissue compartments. Although Zhao et al. showed a negative regulation of PRDM16 by androgens in animals given a nonaromatizable androgen (17), these authors also reported higher levels of PRDM16 in the omental WAT of women compared to men (17) suggesting that the presence of E2 (which is normally found in humans irrespective of gender), overcomes the suppressive effect of androgens on PRDM16. Moreover, an *in-vitro* study by Sul et al. reported that the adipose tissue browning effect of E2 could be mediated by enhanced levels of PRDM16 (18). Our results showed significantly increased levels of circulating E2 and E/T ratio after 6 months of T therapy, likely from enhanced conversion of exogenously administered T to E2. This could account for the increased expression of PRDM16 not only in the SCF, but also in the PBMC and in the serum of men treated with T. We hypothesize that E2 has stimulatory effect on PRDM16 biosynthesis and is responsible for the increase in PRDM16 in all tissues examined in our subjects. While prior studies have established PRDM16 as the crucial switch between white and brown adipogenesis (13), a study by Jiang et al. suggested that it may also function as a switch between adipogenesis and myogenesis (27). In their study, targeted inhibition of PRDM16 by MIR-499 resulted in inhibition of adipogenic while enhancing myogenic markers. Results from our study seem to support the potential role of PRDM16 on the shift from adipogenesis to myogenesis.

Aside from browning of white adipose tissue, it is possible that PRDM16 could have a significant contribution to other aspects of adipose tissue biology. Overexpression of PRDM16 was found to reduce adipogenesis by inhibiting maturation of pig preadipocytes to mature adipocytes through increased lipolysis (28). In this study, there was a significant reduction on PPARγ expression in the early stages of preadipocyte differentiation. Similarly, we found a significant reduction in PPARγ and increase in PRDM16 in the SCF of our subjects which may account for decreased body fat in our hypogonadal men treated with T.

In the myogenic cascade, *PAX7* is the critical transcription factor which regulates regeneration and proliferation of myogenic precursors (29)**;** *MYF5*, is the key transcription factor for myogenic stem cells commitment to myoblastic lineage while *MYOD* directs progenitor cells to myocyte differentiation (30). In preclinical studies, T (100nM) upregulated Pax7 in satellite cells of 2–3-month-old C57BL/6 male mice (23). Another study also reported an increase in the satellite cells of denervated levator ani muscle of rats given T implants compared to non-treated animals (31). Furthermore, Sihna-Hikim et al. demonstrated that T therapy at doses of 300 and 600mg weekly for 20 weeks caused a significant increase in satellite number of the vastus lateralis muscle compared to baseline resulting in muscle hypertrophy (32). The significant increase in PAX7 in the serum of our patients along with the significant increase in *MYOD* expression in PBMC and levels of FST in the serum seem to support the effect of T in stimulating the commitment of skeletal MSCs into the myogenic lineage (33). FST has been reported to increase with T therapy and promote myogenesis by inhibiting MYOSTATIN, a negative regulator of myogenic proliferation (23). Although we cannot detect a significant change in *MYF5* or MYOSTATIN in our study, the small sample size in our study may have impaired our ability to observe such change.

Although our results showing upregulation of MYOD with increase in PRDM16 may contradict previous concept about the function of PRDM16 on myogenesis, Li et al, found that deletion of PR domain from PRDM16 protein in C2C12 cells resulted in significant downregulation of myofiber markers, MyoD, MyHC (Myosin heavy chain) and MCK (Muscle creatinine kinase) as compared to cells transfected with intact PRDM16 (34). The PR domain gene family (PRDM) encodes 19 different transcription factors which are the zinc finger motifs to mediate protein-RNA, protein-DNA interactions (35). In general, PRDM16 exist in two isoforms, a full length PRDM16 with PR domain (fPRDM16) and short PRDM16 which lacks the N-terminal PR domain (sPRDM16) Each isoform has opposing effects, at least in malignancies (38) For instance, while the fPRDM16 is crucial for hematopoetic stem cell (HSC) maintenance and functions as leukemia suppressor, sPRDM16 has the ability to maintain the elongated mitochondria in HSCs, induce inflammation and promote the development of leukemic cells (36). In humans, 1p36 chromosomal deletion that includes the terminal 14 exons of PRDM16 (including exons 4 and 5 of the PR domain) and inactivating point mutations in the gene resulting in loss of function have been found among individuals with left ventricular noncompaction cardiomyopathy and dilated cardiomyopathy (37) The cardiac abnormalities in these patients were attributed to findings of impairment in cardiomyocyte proliferative capacity and even apoptosis of these cells (38) Furthermore, mice with conditional PRDM16 knockout in vascular smooth muscle cell showed increased apoptosis, while PRDM16 deficiency has been demonstrated in abdominal aortic aneurysm lesions in humans (39). Although the latter are examples of pathology involving smooth muscles, yet in conjunction with the data from Li et. al., perhaps would altogether suggest a positive role of PRDM16 in myogenesis (skeletal and smooth muscles). On the other hand, it is also possible that *PRDM16* may upregulate the myogenic pathway via unknown downstream factors. As far as our study is concerned, we measured the full-length PRDM16 transcripts in the SCF and PBMC and the full-length protein in the serum. By showing a correlation between changes in PRDM16 protein levels in the serum and changes in lean and fat-free mass, our data suggest that PRDM16 could have a distinct and separate role in promoting myogenesis apart from its well-recognized function as the adipomyogenic switch.

The strengths of our study include: 1) novelty, our study is the first to explore gene and protein machinery involved in the increase in lean mass and reduction in fat mass in men with hypogonadism given T therapy, and 2), this is the first study to evaluate the molecular mechanism behind the positive effects of T-therapy *in-vivo* on body composition in hypogonadal men. However, this study presents several limitations. Firstly, it is a secondary analysis of samples from our prior clinical trial; hence, we have limited number of samples available to perform longitudinal analysis of gene and protein expression that occur with T therapy and this may have resulted in lack of significance in certain adipogenic and myogenic markers. Secondly, PBMC was used as a surrogate of skeletal muscle gene expression. However, gene expression studies in PBMCs can be used as a substitute for muscle gene expression has been suggested by Rudkowska et al. (21). These authors investigated the correlation between transcriptome in PBMCs and skeletal muscle tissues before and after 8-week supplementation with n-3 polyunsaturated fatty acid (PUFAs) in 16 obese and insulin resistant human subjects (7). They found that 88% of the transcripts were co-expressed in both tissues. Moreover, there was a strong correlation (*r* = 0.84, *p* < 0.0001) between transcript expression levels in PBMCs and skeletal muscle tissues after *n*−3 PUFA supplementation. These led them to conclude that in the interest of cost and practicality, PBMCs can be used as a surrogate model for skeletal muscle gene expression. Furthermore, very recently Banerji et al. (2023) found that expression of PAX7 target genes in the muscle of fascioscapulo humeral muscular dystrophy patients (the so-called FSHD blood-biomarker) was significantly correlated with the levels in PBMCs (P=0.002) and suggested this could be used as a novel muscle-blood biomarker to measure clinical severity of FSHD (40), again suggesting the potential utility of PBMCs as surrogate for muscles. Lastly, both Prdm16 and Pax7 are traditionally considered as nuclear proteins. However, newer data suggests that nuclear contents can be detected outside of the nucleus and inside extracellular vesicles (EVs) (41). EVs carry proteins and can transfer from one tissue to another or enter the circulation allowing tissue to tissue communication (41, 42). The presence of PRDM16 in EVs has been reported by Sun et.al (43). These investigators showed that uptake of PRDM16-containing EVs from hepatic stellate cells by hepatocellular carcinoma cells results activation of NOTCH signaling leading to tumor progression (43). Thus, presence of PRDM16 in EVs may allow its detection in the serum as our study was able to determine. Similarly, for PAX7, it is possible that EVs carrying PAX7 find their way into the circulation allowing measurement of this factor in the serum. Nevertheless, since we are the first to report measurement of both factors in the serum, our findings need confirmation in future studies.

## 5 Conclusion

In summary, our results suggest that T therapy promoted a shift from the adipogenic to the myogenic gene/protein machinery which may explain the reduction in body fat and increase in lean mass in our subjects of older hypogonadal men. These desirable effects on body composition may be modulated in part by enhanced PRDM16 driven signal resulting from an increase in estradiol levels leading to subsequent changes in downstream target of genes and proteins involved in adipogenesis and myogenesis. Thus, PRDM16 may represent a target for drug development to improve body composition in patients with age-related loss in muscle mass with concomitant increase in fat mass such as those with sarcopenic obesity.

## Data Availability

The original contributions presented in the study are included in the article/supplementary material, further inquiries can be directed to the author RA-V.
