# Supplementary material for: A PRDM16-driven signal regulates body composition in testosterone-treated hypogonadal men
Source: Front Endocrinol (Lausanne). 2024 Sep 2;15:1426175. doi: 10.3389/fendo.2024.1426175 (PMC11402695; doi:10.3389/fendo.2024.1426175)
Supplement: Supplementary file 1 [file DataSheet1.pdf]

SUPPLEMENTARY TABLE: KEY RESOURCES

REAGENT/RESOURCE	SOURCE	IDENTIFIER
**Chemicals, Peptides, Metabolites, and Recombinant Proteins**		
RNALater T	ThermoFisher	Cat# AM7020
HPLC-grade water	ThermoFisher	Cat# W64
Testosterone-2H3	Sigma-Aldrich	Cat# T2655
2-Mercaptoethanol	Sigma-Aldrich	Cat# M6250
Halt Protease Inhibitor Cocktail (100X)	ThermoFisher	Cat# 78430
HEPES	Biotechne	Car# 7365-45-9
PBS	Corning	Cat# 21-040
**Critical Commercial Assays/Machines**		
RNeasy Plus Universal Mini Kit	QIAGEN	Cat# AM1928
TaqMan Universal PCR Master Mix	ThermoFisher	Cat# 4304437
SuperScript VILO Master Mix	ThermoFischer	Cat# 11755250
96 well plate for PCR	Applied Biosystems	Cat# N8010560
FastPrep 24–5G homogenizer	MP Biomedicals	Cat # 1606-1026
Nanodrop and Bioanalyzer 2100	Agilent Technologies	Cat# P 275
Real Time PCR system	Applied Biosystem-	Cat # F272520135
Accuwash	ThermoFisher	Cat# 888-7482AF
Digital incubator	Wards science	Cat # 03211213
Spectra max Ab plus	Molecular devices	Cat# ABP 00603
Human Adiponectin ELISA kit	R&D	Cat# EZHADP-61K
Human Leptin ELISA kit	R&D	Cat # EZHL-805K
Human Follistatin ELISA kit	R&D	Cat # DFN00
Human GDF-8/Myostatin Immunoassay	R&D	Cat# DGDF80
Human CFD (Adipsin) Elisa kit	ThermoFisher	Cat# EHCFD
Human PRDM16 Elisa kit	Biomatik	Cat# EKN52948
Human PAX7 Immunoassay kit	My Biosource	Cat# MBS2606278
**Oligonucleotides**		
Taqman FAM Probe PPARγ	ThermoFisher	Hs01115513_m1
Taqman FAM Probe CEBPα	ThermoFisher	Hs00269972_s1
Taqman FAM Probe LPL	ThermoFisher	Hs00173425_m1
Taqman FAM Probe PAX7	ThermoFisher	Hs00242962_m1
Taqman FAM Probe PRDM16	ThermoFisher	Hs00223161_m1
Taqman FAM Probe MYF5	ThermoFisher	Hs00929416_g1
Taqman FAM Probe MYOD	ThermoFisher	Hs00159528_m1

Taqman VIC Probe -18s	ThermoFisher	Hs03928990_g1
**Software and Algorithms**		
Quantstudio design and analysis software 1.3.1	ThermoFisher	N/A
Endnote version 21	Clarivate Analytics	N/A
Biorender software	Toronto, Ontario, Canada	N/A
Graph pad prism software 9.0	Dotmatics, CA, USA	N/A
